# Seasonal assessment on the effects of time of night, temperature and humidity on the biting profile of *Anopheles farauti* in north Queensland, Australia using a population naive to malaria vector control pressures

**DOI:** 10.1186/s12936-023-04495-5

**Published:** 2023-03-08

**Authors:** Weng K. Chow, Nigel W. Beebe, Luke Ambrose, Paul Pickering, Robert D. Cooper

**Affiliations:** 1 Australian Defence Force Malaria and Infectious Disease Institute, Gallipoli Barracks, Enoggera, QLD Australia; 2 School of Biological Sciences, The University of Queensland, St Lucia, QLD 4072 Australia; 3 CSIRO, Dutton Park, QLD 4102 Australia

**Keywords:** Malaria, *Anopheles farauti*, Biting behaviour, Behavioural insecticide resistance, Circadian clock

## Abstract

**Background:**

*Anopheles farauti* is one of the major vectors of malaria in the Southwest Pacific region and is responsible for past outbreaks in Australia. With an adaptable biting profile conducive to behavioural resistance to indoor residual spraying (IRS) and insecticide-treated nets (ITNs), its all-night biting behaviour can switch to biting mostly in the early evening. With limited insight into the biting profile of *An. farauti* populations in areas that have not encountered IRS or ITNs, the aim of this study was to develop insights on the biting behaviour of a malaria control naive population of *An. farauti.*

**Methods:**

Biting profiles of *An. farauti* were conducted at Cowley Beach Training Area, in north Queensland, Australia. Initially, encephalitis virus surveillance (EVS) traps were used to document the 24-h biting profile of *An. farauti* and then human landing collections (HLC) were used to follow the 18.00–06.00 h biting profile. The human landing catches (HLC) were performed at both the end of the wet (April) and dry (October) seasons.

**Results:**

Data exploration using a Random Forest Model shows that time of night is the most important variable for predicting *An. farauti* biting activity. Temperature was found to be the next important predictor, followed by humidity, trip, collector, and season. The significant effect of time of night and peak in time of night biting, between 19.00 and 20.00 h was also observed in a generalized linear model. The main effect of temperature was significant and non-linear and appears to have a positive effect on biting activity. The effect of humidity is also significant but its relationship with biting activity is more complex. This population’s biting profile is similar to populations found in other parts of its range prior to insecticide intervention. A tight timing for the onset of biting was identified with more variation with the end of biting, which is likely underpinned by an endogenous circadian clock rather than any light intensity.

**Conclusion:**

This study sees the first record of a relationship between biting activity and the decreasing temperature during the night for the malaria vector, *Anopheles farauti*.

**Supplementary Information:**

The online version contains supplementary material available at 10.1186/s12936-023-04495-5.

## Background

Throughout the Southwest Pacific region where malaria occurs, one of the primary vectors is *Anopheles farauti* [1]. It has a wide distribution, being found from the Moluccas (Indonesia) in the west, throughout Papua New Guinea (PNG), Solomon Islands, Vanuatu and northern Australia. *Anopheles farauti* is a coastal species rarely found more than 5 km from the coast with brackish swamps being the preferred oviposition site, although it is commonly found in fresh water sites [2].

With regard to malaria transmission, *An. farauti* has a reputation for being difficult to control in the Solomon Islands using the traditional malaria vector control methods of indoor residual spraying (IRS) [3–5] and insecticide-treated nets (ITNs). The feeding behaviour of this species, documented by several studies before the introduction of DDT IRS, shows *An. farauti* starting to feed at dusk and progressing to a peak around midnight: in the D’Entrecasteaux Islands (PNG) [6], New Britain (PNG) [7] and the Solomon Islands [8, 9]. Following the introduction of DDT IRS into PNG and the Solomon Islands during the 1960s and 1970s, *An. farauti* shifted its peak biting time forward to between 18.00 and 20.00 h. In New Britain (PNG), after five spray cycles (across two years) with DDT IRS, there was a distinct shift in the peak biting time from the middle part of the night to between 18.00 and 19.00, with 76% of feeding occurring before 21.00 [7]. Similar studies in the Solomon Islands also saw a shift in biting activity moving from midnight to an early night peak followed by a second peak just before sunrise [8, 9].

More recent studies in the Solomon Islands confirm the early night biting peak occurring before 21.00 remains in these populations despite the cessation of DDT spraying in the 1980s [5, 10]. The lack of a reversion to the original phenotype of biting through the night after the IRS selection pressure ended is interesting as there would be populations of *An. farauti* extant on the islands where malaria control activities did not take place. However, a possible reversion did occur on Buka Island (PNG) where 19 years after the DDT IRS control campaign (1961–1980) ceased, the *An. farauti* population exhibited a peak biting time of 00.00–01.00 [11, 12].

Molecular identification methods suggest *An. farauti* in the Solomon Islands to be a single species [4, 13, 14], and so it remains unclear why these populations would not revert back to an original feeding phenotype after the Global Eradication Campaign in the 1970s in the Solomon Islands, but could possibly be attributed the strong selection on small island populations. More recent use of long-lasting insecticidal nets (LLINs) has likely reinforced this early evening biting behaviour [10, 15]. A better understanding of the feeding behaviour of *An. farauti* in malaria control naive populations may shed some light on this phenomenon.

Despite the use of LLINs in the Southwest Pacific, malaria has been increasing since 2015 with the largest increases seen in the Solomon Islands [16]. Communities in PNG and the Solomon Islands spend much of the early part of the night outdoors [15, 17], and *An. farauti*, by feeding early, can obtain a bloodmeal without entering houses. Thus, these shifts in peak feeding suggest changes in mosquito biting behaviour to avoid the insecticide, resulting in uncontrolled outdoor malaria transmission. Additionally, day time biting has been recorded with *An. farauti* [18, 19] using collections from 07.00 to 10.00 [19]; although the numbers collected were small, no specific attempt was made to assess day time biting activity during these early studies.

Unlike PNG and the Solomon Islands, malaria on mainland Australia is not a serious problem [20]. Thus, DDT IRS was never employed for malaria control and anophelines in Australia have not encountered sustained indoor insecticide selection pressures. *Anopheles farauti* is thought to be the main vector of malaria in Australia [21], with the last outbreak by this mosquito being *Plasmodium vivax* in 2002 [22]. The host-seeking behaviour and night-biting profile of this species in Australia is not well understood. Additionally, there are other aspects of the host-seeking behaviour of *An. farauti* that have not been studied in any part of its range, such as the exact time host seeking commences and then ceases. Traditionally, human landing catches (HLC) commence at 18.00 and finish at 06.00, but does host seeking occur before and after these times? What triggers this host-seeking behaviour, does the relationship between light intensity and times of biting in relation to sunset and sunrise have an effect? There are limited studies to date that has explored how light can affect biting behaviour. Also, what effect does temperature and humidity have on host seeking?

The aim of this study is to determine the biting profile of a population of *An. farauti* that has not encountered indoor malaria-control based insecticide treatments. It was hypothesized that the populations in north Queensland will show host-seeking activities throughout the night with a peak around midnight as has been found in other *An. farauti* populations prior to indoor insecticide pressure.

## Methods

### The study site

**Fig. 1:**
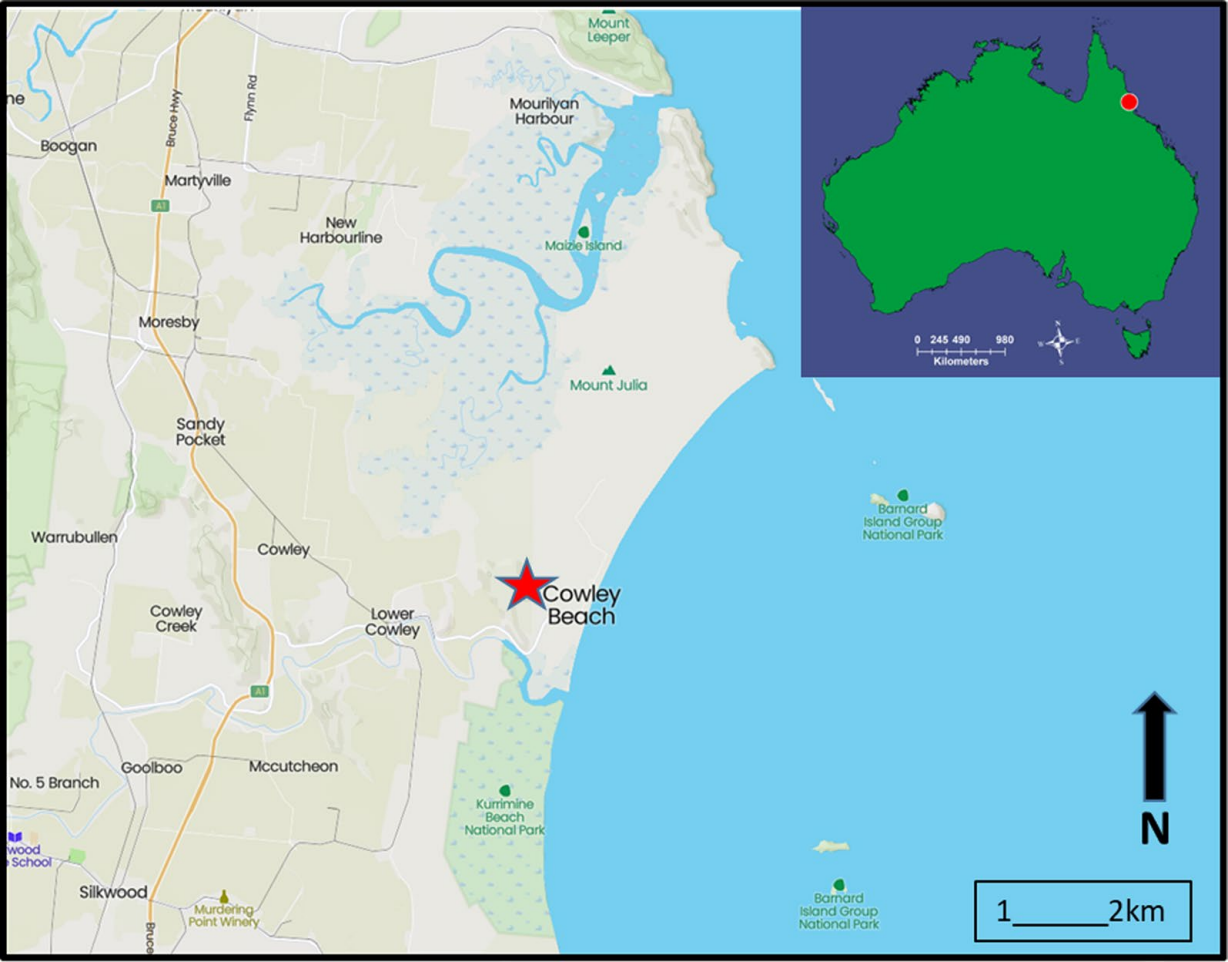
Study location and collection site at Cowley Beach Train Area, north Queensland, Australia (17° 41′ 14.7″ S 146° 06′ 06.4″ E)

Collections of *An. farauti* were made at the Cowley Beach Training Area (CBTA), an Australian Defence Force training facility, situated in the wet tropical region in far north Queensland, Australia, approximately 40 km south of Innisfail (Fig. 1). The training area comprises 5081 ha of coastal lowland plains consisting of mixed open forest, melaleuca swamps, estuarine aquatics, rainforest and 8 km of beaches [23]. Tidal flats, potential *An. farauti* oviposition sites, cover approximately 37% of CBTA and consist mainly of regularly inundated areas with mangroves and tidal creeks [23].

The climate type for the area is monsoonal with a distinct wet (November to April) and dry (May to October) season. There is seasonality in the temperature with May to October being the cooler months and November to April the hotter months with mean minimum and maximum temperatures of 15.8 °C to 28.0 °C and 21.1 °C to 30.7 °C, respectively. Innisfail receives a mean annual rainfall of 3500 mm per year (n = 139 years), making it one of the wettest places in Australia. The mean relative humidity is high (75–80%) and is constant throughout the year [24].

### Biting activity

*Anopheles farauti* biting activity was determined by HLC, a total of 25 nights of collection were conducted at the end of the wet season (April) and 34 nights of collection conducted at the end of the dry season (September, October) from 2014 to 2017. Collections were made at a single site (17° 41′ 14.7″ S 146° 06′ 06.4″ E) 1.4 km inland from the coast and at the boundary of mixed open forest and rainforest 1.5 km south of the primary oviposition site (Fig. 1). During HLC, the collector exposed their lower legs (between ankle and knee) to host-seeking mosquitoes for 50 min each hour from 18.00 to 06.00. Catches were made by two collectors, one collector working from 18.00 to 00.00 and the second collector working from 00.00 to 06.00. The collectors rotated between shifts throughout the collection periods to reduce bias for differences in individual odours and collecting abilities. The collectors monitored for mosquitoes that landed on their legs; using a torch, any anophelines landing were captured using a mouth aspirator. The mosquitoes were then placed into a wax-lined paper cup, covered with mosquito netting labelled for each hour of the collections. Anophelines collected were killed by freezing and the numbers caught for the hour were counted and recorded. To determine if temperature or humidity affected the biting profile, these parameters were recorded on the hour for each hour of collection. To determine the 24-h biting profile, an encephalitis virus surveillance (EVS) trap [25] was set for 24 h at the collection site. The trap was collected each hour and the number of *An. farauti* collected was counted and recorded. This was replicated over four nights.

### Species identification

All *Anopheles* collected were initially identified by adult morphology to the *An. farauti* complex [26]. As two other isomorphic species: *Anopheles hinesorum* and *Anopheles torresiensis* are known to occur in north Queensland coastal region [27], sub-sets of anophelines from each collection period were preserved in 70–100% ethanol for transport to the laboratory where they were assessed by PCR-based diagnostics to confirm the target study species was *An. farauti* [28].

### Factors associated with biting profiles

To determine the commencement of biting, the time was recorded when the first *An. farauti* was collected. To determine the cessation of biting, collections were continued after 06.00 and until 20 min had elapsed since the last *An. farauti* was collected. At each of these time points the temperature, humidity and lumination intensity (lux) (Lutron light meter, Model LX-1108) was recorded.

In order to assess the most important variables recorded that may be associated with the biting activity of *An. farauti*, data exploration was initially performed to assess overall patterns in the data. As time of night is known to be important in predicting the biting activity of this species, the mean and standard errors of numbers of *An. farauti* collected in both the wet and dry seasons were plotted. As expected from previous studies, it was shown that there is a non-linear trend in data associated with time of night (Additional file 1: Fig. S1). A Generalized Additive Model (GAM) was fitted to the data to further assess the effects of the other recorded variables, as well as to account for potential effects of collector and temporal factors. This was performed using the gam function in the R package [29] ‘mgcv’ [30]. Also initially fitted was a Random Forest Model using the R package ‘randomForest’ [31], to assess which variables are likely to be the strongest predictors of mosquito biting activity. Based on the results of data exploration, the final GAM that was fit to the data was Number ~ s(Time, k = 12) + s(Temperature) + ti(Time, Temperature, k = 12) + s(Humidity) + s(Date, k = 7, bs = “re”) + Collector.

## Results

### Species identification

All mosquitoes collected by HLC during the wet and dry seasons (n = 18,006) were determined to be *An. farauti s.l.* by morphology. A sub-set of the specimens was confirmed as *An. farauti* by molecular analysis (n = 250), with no other cryptic species present.

### Time of night, temperature, humidity and other predictors of *An. farauti* biting activity

**Fig. 2:**
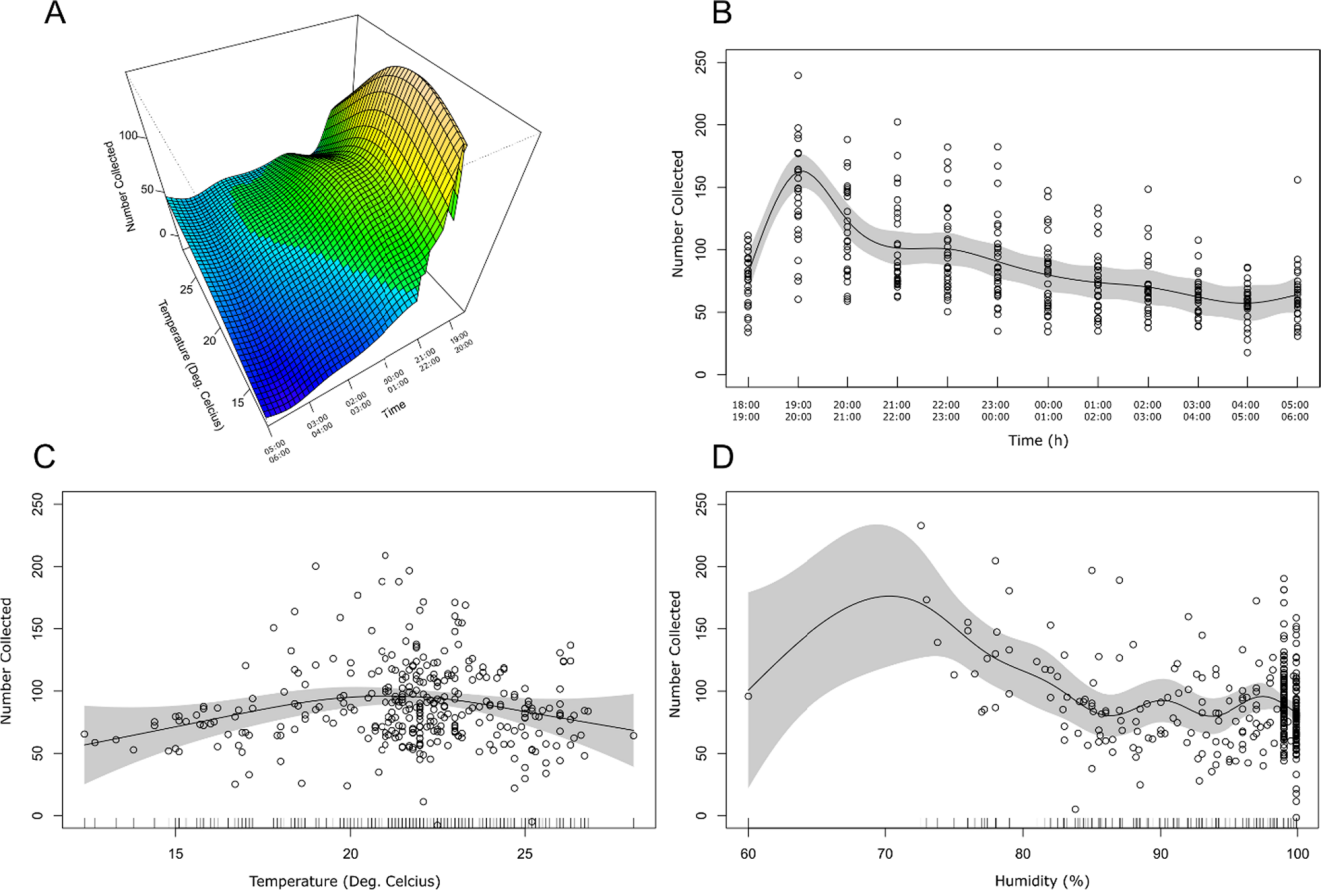
Smooths from the Generalized Additive Model. **A** Surface plot of combined effects of temperature and time of night on number of *An. farauti* collected. **B** Partial smoothed effect of time of night on number of *An. farauti* collected. **C** Partial smoothed effect of temperature on number of *An. farauti* collected. **D** Partial smoothed effect of humidity on number of *An. farauti* collected. Residuals and standard errors are shown in B-D

**Fig. 3:**
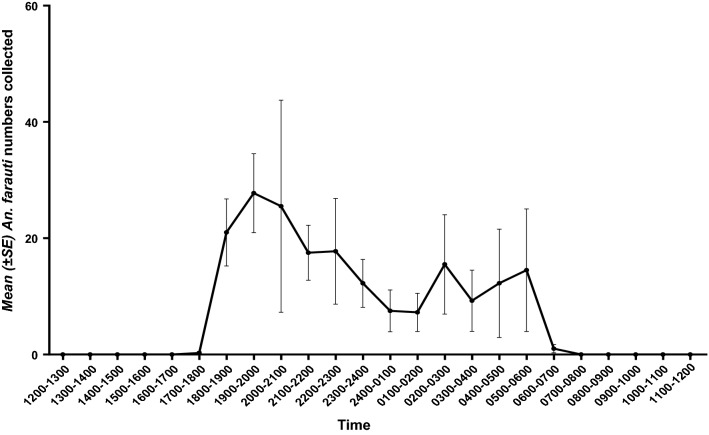
*Anopheles farauti* mean feeding activity (± SE) from EVS traps for 24 h over four days at CBTA, Australia shows highly nocturnal biting behaviour

At CBTA, *An. farauti* commenced feeding early in the night, peaking between 19.00 and 20.00, biting activity then fell off over the remainder of the night (Fig. 2 and Additional file 1: Fig. S1). In the dry season (April), the majority of *An. farauti* biting activity occurred before midnight (76%) with two biting peaks, the highest at 19.00–20.00 and a lesser peak at 22.00–23.00 (Fig. 2A and Additional file 1: Fig. S1). Similarly, in the wet season (October), 64% of the biting activity occurred before midnight with a biting peak also at 19.00–20.00 (Additional file 1: Fig. S1). A highly fixed timing for the commencement and cessation of biting was found between collection nights (Table 1). Additionally, the light intensity observed for the commencement and cessation of biting was highly consistent in both the wet and dry seasons (Table 1). Overall, light intensity at the cessation of biting in the morning was greater than that at start of biting in the evening. The four outdoor 24-h EVS collections (n = 756) made at CBTA at the collection site suggested *An. farauti* were not caught between the times of 06.00 and 18.00 (Fig. 3). The biting numbers (total) of *An. farauti* were higher in the dry (n = 10,821) than the wet (n = 7185) seasons.

**Table 1:** *Anopheles farauti* commencement and cessation time of biting and lumination results during the wet and dry seasons at CBTA, Australia

Season	Wet	Dry
Temperature (°C)	23 ± 1 (min 15–max 28) (n = 14)	20 ± 1, (min 12–max 26) (n = 15)
Humidity (%)	96 ± 1 (n = 14)	95 ± 1 (n = 15)
Time of first bite	1832 h ± 3 min (n = 25)	1835 h ± 1 min (n = 34)
Lumination (Lux)	1.7 ± 1.1 (n = 22)	2.5 ± 1 (n = 22)
Time of last bite	0617 h ± 6 min (n = 25)	0546 h ± 4 min (n = 34)
Lumination (Lux)	225.4 ± 147 (n = 22)	144 ± 19 (n = 22)

n: number of collection nights

**Fig. 4:**
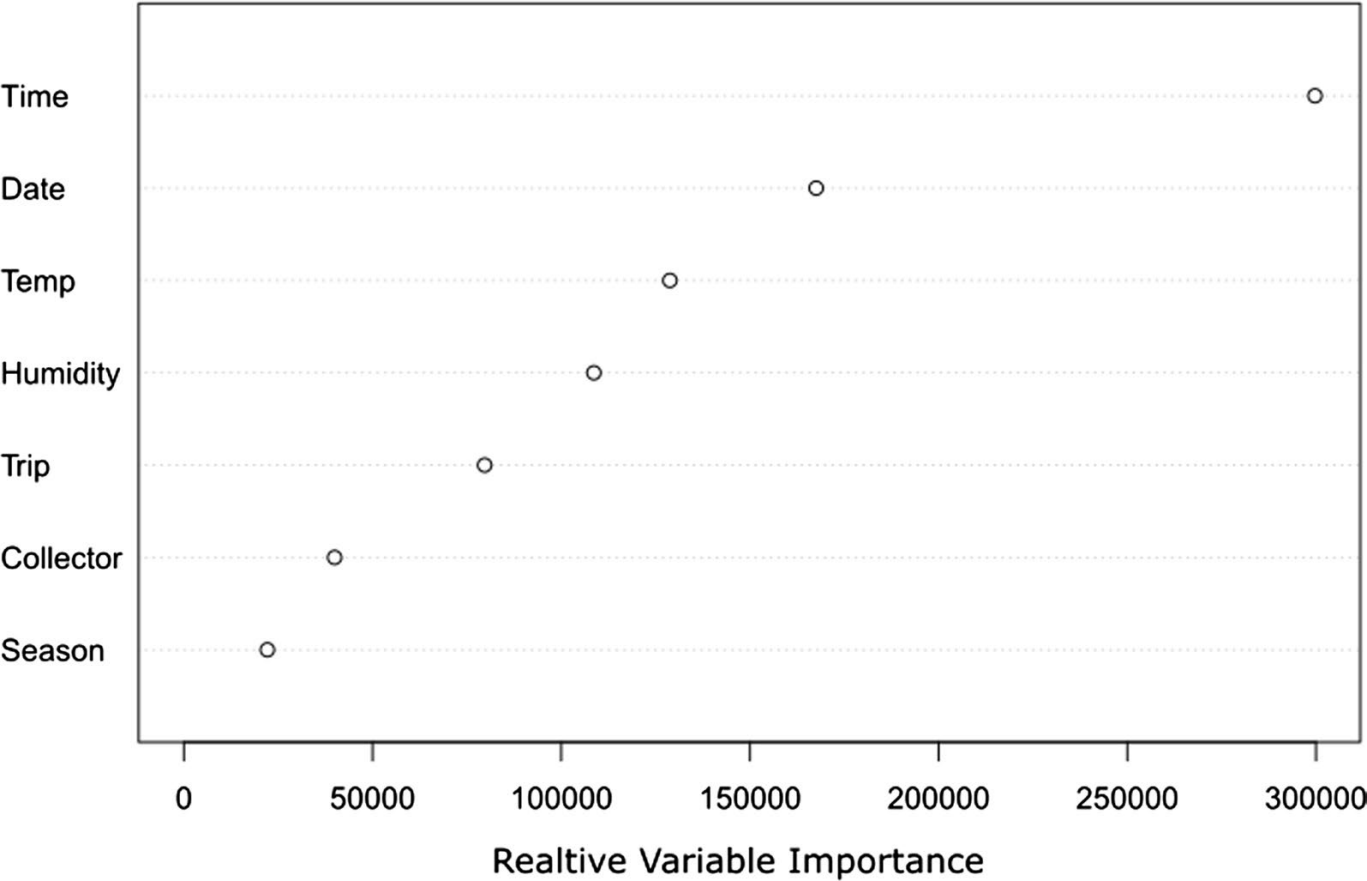
Random Forest Model assessing relative importance of predictor variables

Data exploration using a Random Forest Model shows that time of night is clearly the most important variable measured for predicting *An. farauti* biting activity (Fig. 4). This is followed by the temporal variable, date of which likely reflects fluctuations in the numbers of mosquitoes caught due to other environmental factors not recorded (such as rainfall and wind). Thus, date was included in the GAM as a random effect. Temperature was found to be the next most important predictor, followed by humidity, trip, collector, and season. Since any temporal autocorrelation associated with differences in number of mosquitoes collected should be accounted for by date, trip was not included as a factor in the GAM. Likewise, season was excluded due to both its low predictive power and obvious collinearity with temperature and humidity. The final GAM that was fit to the data had an adjusted R squared of 0.472 and explained 50.9% of the deviance in residuals. All non-random variables fitted were significant and non-linear, with time of night having the most complex relationship with mosquito biting activity (edf = 9.73) as well as being the most significant predictor (P < 2e−16). To visualize these complex non-linear effects, the smooths fitted to the data are presented, as well as the standard errors and means associated with them (Fig. 2). Collector was fit as a parametric (linear effect) allowing the intercept of collector to vary in the model. From this, there were found significant differences in intercept between the two collectors (P = 0.0001, t = − 3.877), with a mean difference of 16.44, after adjusting for other variables. The significant effect of time of night and peak in time of night biting, between 19.00 and 20.00, can be clearly seen in Fig. 2A and B. The main effect of temperature was significant (P = 0.006) and non-linear (edf = 2.36) and appears to have a positive effect on biting activity between 12.4 °C and approximately 22 °C, after adjusting for other variables, above which it has a negative effect when considered as a main effect (Fig. 2C). However, there is also a clear interaction between time of night and temperature, as indicated by the highly significant and non-linear tensor interaction term (P = 6.93e−0.5, edf = 2.46). From Fig. 2A, it can be seen that this interaction predicts that on warmer nights, the biting peak between 19.00 and 20.00 is less pronounced, and the biting profile appears flatter, with mosquitoes being collected more consistently through the evening. The effect of humidity is also significant (P = 0.0002) but its relationship with biting activity is more complex (edf = 7.38) and difficult to interpret (Fig. 2D).

The four coldest and four warmest nights during the study were used to assess the effect of temperature on biting activity. Cold night average temperatures were below 17 °C and warmest nights had average temperatures above 25 °C. The cooler nights occurred during the dry season (spring) when *An. farauti* densities numbers were higher (n = 2411) and the decreasing temperature throughout the night reduced biting activity. In this, a temperature threshold of 18 °C saw *An. farauti* biting activity drop rapidly (Additional file 1: Fig. S2). On warmer nights (n = 4) of collection (n = 1957 *Anopheles* collected), the temperature was consistent throughout the night and *An. farauti* biting activity peaked between 19.00 and 01.00 (Additional file 1: Fig. S2).

## Discussion

Accurate knowledge of the biting profile or the night biting profile of malaria vectors is important as the two main control strategies currently used against malaria vectors remain controlling the host-seeking females with IRS and LLINs. For these measures to be effective, the vector should be seeking a bloodmeal when humans are either indoors and/or under an ITN. Under these conditions the vector should come into contact with the insecticide, either on the walls or the net and die. If the vector feeds before this time, it will likely feed outdoors and avoid contact with insecticide and outdoor transmission will continue despite the intervention measures.

This study of *An. farauti* at CBTA was carried out over four years in both wet (April) and dry (October) seasons. The night biting profile of this *An. farauti* population, which has not been subjected to IRS or ITNs appears to show a similar biting profile to other population that were subjected to insecticidal pressure IRS or ITNs [5, 7, 32]., When more extreme ambient temperature is considered, a decreasing temperature below 18 °C through the night reduces biting activity with most activity limited to the warmer early evening window. On warmer nights (above 25 °C), where the temperature is constant, biting appears to occur through the night, similar to other populations in the region that had not been subjected to indoor insecticidal pressure, as in pre-spray PNG [12, 19, 33] and the Solomon Islands [8, 32]. In this, the mean temperature in the equatorial coastal lowlands of PNG and the Solomon Islands is around ~ 25 °C (min 22 °C-max 31 °C) [34] and biting activity does not usually occur at temperatures below 20 °C. The observation in Queensland, a population at a higher latitude and thus encountering a broader temperature fluctuation, sees the first record of a relationship between a reduction in the biting activity of *An. farauti* and decreasing temperature during the night. Mosquitoes are ectotherms and metabolism is dictated by ambient temperature [35, 36], with tropical mosquitoes sensitive to lower ambient temperatures affecting flight and biting activity. Temperature and biting activity studies on another tropical mosquito also found in Queensland, Australia, *Aedes aegypti*, suggests biting activity decreases and can even stop at between 15 °C and 18 °C [37] and may help understand the biting profile of *An. farauti* on these cooler nights.

*Anopheles farauti* shows a tight coastal distribution with the ability to utilize both brackish and fresh water larval sites [2, 38]. This mosquito can display higher population densities during the dry season as observed in studies in the Solomon Islands [10, 39]. Also seen is a similar seasonal influence in the CBTA study site, where adult productivity is higher during the dry season and this pattern is probably due to larvae being flushed out to the sea by heavy tropical rainfall during the wet season.

Ecological niche modelling of *An. farauti* in Australia found a strong correlation between parameters of temperature (i.e., minimum, diurnal, seasonal and annual temperature range as well as sea temperature), which, apart from sea temperature, are moderated along the coast by the sea temperature [40]. The observed decrease in female host-seeking activity with cooler ambient temperatures complements a similar finding [40], that these environmental parameters may help define the range for *An. farauti* and may prevent the species moving inland where larger temperature variation would be encountered, although temperature fluctuations decrease in more equatorial regions.

The tight feeding timing consistency appears more tied to a 24-h clock rather than light intensity and complements the findings of Duffield et al. [41], in that *An. farauti* daily nocturnal flight activity is underpinned by the circadian clock—a research area in mosquitoes now beginning to unfold with new genomic and transcriptomic tools available [42–45].

## Conclusion

*Anopheles farauti* is one of the most important malaria vectors in the Southwest Pacific and has shown consistent behavioural resistance to indoor malaria control strategies such as IRS and ITNs, with populations moving to earlier evening biting under this selection pressure.

This population displays biting time plasticity with early biting preference observable when night temperature drops below 18 °C to biting through the night on warmer nights that maintain temperatures above 25 °C. It would be interesting to consider how this feeding time plasticity is controlled and if there is any connection with observed behavioural insecticide resistance in this malaria vector.

## Supplementary Information

**Additional file 1: Fig. S1.** *Anopheles farauti* mean (± SE) biting profile from HLC at CBTA for 15 nights each during the wet (Apr) and dry (Oct) seasons between 2014 and 2017. **Fig. S2.** (A) *Anopheles farauti* mean biting profile on four of the coldest nights during the study. (B) *Anopheles farauti* mean biting profile on four of the hottest nights during the study. Mean (± SE) *An. farauti* collected per person (black) compares with temperature (red).

## Data Availability

The datasets generated and analysed during the current study are available through DRYAD DOI https://doi.org/10.5061/dryad.s1rn8pkc2 and contains a README file and mosquito trapping results in an.xlsx file.

## Abbreviations

IRS: Indoor residual spraying
ITN: Insecticide treated nets
EVS: Encephalitis virus surveillance trap
HLC: Human landing catch
PNG: Papua New Guinea
LLIN: Long-lasting insecticidal net
CBTA: Cowley Beach Training Area
ADFMIDI: Australian Defence Force Malaria and Infectious Disease Institute
