# Supplementary material for: Seasonal assessment on the effects of time of night, temperature and humidity on the biting profile of Anopheles farauti in north Queensland, Australia using a population naive to malaria vector control pressures
Source: Malar J. 2023 Mar 8;22:85. doi: 10.1186/s12936-023-04495-5 (PMC9996873; doi:10.1186/s12936-023-04495-5)
Supplement: Supplementary file 1 — Additional file 1: Fig. S1. Anopheles farauti mean (± SE) biting profile from HLC at CBTA for 15 nights each during the wet (Apr) and dry (Oct) seasons between 2014 and 2017. Fig. S2. (A) Anopheles farauti mean biting profile on four of the coldest nights during the study. (B) Anopheles farauti mean biting profile on four of the hottest nights during the study. Mean (± SE) An. farauti collected per person (black) compares with temperature (red). [file 12936_2023_4495_MOESM1_ESM.docx]

**Supplementary figures**

**Fig. S1**  ***Anopheles farauti* mean (±SE) biting profile from HLC at CBTA for 15 nights each during the wet (Apr) and dry (Oct) seasons between 2014 and 2017**


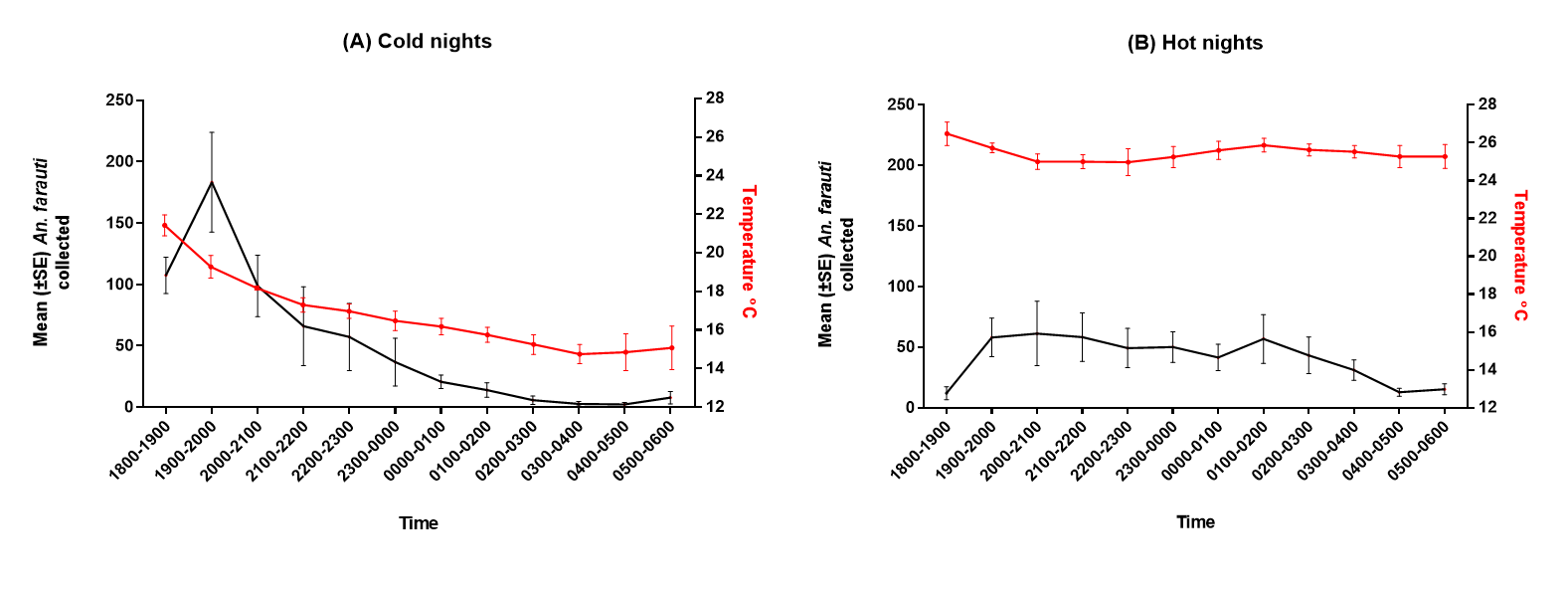


**Fig. S2** **(A) *Anopheles farauti* mean biting profile on four of the coldest nights during the study. (B) *Anopheles farauti* mean biting profile on four of the hottest nights during the study**

Mean (±SE) *An. farauti* collected per person (black) compares with temperature (red).
